# Nanoscale elastic modulus of single horizontal ZnO nanorod using nanoindentation experiment

**DOI:** 10.1186/1556-276X-7-146

**Published:** 2012-02-21

**Authors:** Muhammad Yousuf Soomro, Ijaz Hussain, Nargis Bano, Esteban Broitman, Omer Nur, Magnus Willander

**Affiliations:** 1 Department of Science and Technology, Campus Norrköping, Linköping University, Norrköping, SE-60174, Sweden; 2 Department of Physics, Chemistry, and Biology (IFM), Linköping University, Linköping, SE-58183, Sweden

## Abstract

We measure the elastic modulus of a single horizontal ZnO nanorod [NR] grown by a low-temperature hydrothermal chemical process on silicon substrates by performing room-temperature, direct load-controlled nanoindentation measurements. The configuration of the experiment for the single ZnO NR was achieved using a focused ion beam/scanning electron microscope dual-beam instrument. The single ZnO NR was positioned horizontally over a hole on a silicon wafer using a nanomanipulator, and both ends were bonded with platinum, defining a three-point bending configuration. The elastic modulus of the ZnO NR, extracted from the unloading curve using the well-known Oliver-Pharr method, resulted in a value of approximately 800 GPa. Also, we discuss the NR creep mechanism observed under indentation. The mechanical behavior reported in this paper will be a useful reference for the design and applications of future nanodevices.

## Introduction

Since the last two decades, several kinds of nanomaterials have attracted much interest as nano-building blocks for various fascinating applications. There are many types of nanomaterials such as nanotubes, nanorods, nanobelts, etc. with potential as the future blocks for building different kinds of nanodevices in electronics, optics, and nanoelectromechanical systems [NEMS]. Usually, when nanomaterials are used for nanodevices, they will probably be deformed to some extent. So, clearly, a precise and better understanding of the mechanical properties of a nanostructure is important before this nanoscale material can be successfully incorporated into assembly of nanodevices.

Zinc oxide [ZnO] is a direct, wide-bandgap, II-IV, n-type semiconductor with remarkable physical properties such as being semiconductor, piezoelectric, and biocompatible. Various common forms of quasi-anisotropic, inorganic, one-dimensional [1D] ZnO nanostructures such as nanowires, nanorods, nanobelts, nanorings, and nanohelices have been synthesized by different techniques [1]. Among these structures, 1D nanorods [NRs] have attracted considerable interest in recent years because they exhibit extraordinary properties different from those found in bulk ZnO materials due fundamentally to their high surface-to-volume ratio, unique structural, and quantum confinement effects. These 1D NRs are believed to be useful building blocks and present the utmost challenge to myriad semiconductor technology, making fascinating novel devices like nanoscale interconnects [2], active components of optical electronic devices [3], field effect transistors [4], nanocantilevers [5], and nanogenerators [6].

As mechanical reliability, to some extent, will determine the long-term stability and performance for many of the nanodevices, it is a key parameter to understand the mechanical characteristics of these ZnO NRs prior to any feasible applications. Although a number of pioneering works have been focused on predicting the mechanical properties of nanostructures [7-9], to the best of our knowledge, few available reports on the mechanical properties of ZnO NRs can be found in the literature [10-19]. The elastic modulus, an important mechanical parameter for describing 1D systems, has been reported by different authors with widely scattered values between 30 and 250 GPa, depending on the nature of different experimental techniques.

Atomic force microscopy [AFM] three-point bending and nanoindentation are the most important methods to calculate the elastic modulus of ZnO NRs. The main difference between the AFM three-point bending test and the nanoindentation is that the former technique involves more surface atoms while the latter probes less surface atoms, and the surface atoms are more easily stretched than the atoms locked in the crystal lattice [20]. Nanoindentation has become the most popular, simple, and standard technique for the measurement of the elastic modulus of small samples. In this work, we measured Young's modulus of single ZnO NRs by the three-point bending configuration using the nanoindentation technique.

## Experimental details

The ZnO NRs used in this experiment were synthesized by a simple and a template-free hydrothermal method on n-type silicon substrate. All the chemicals used were of analytical grade from Sigma-Aldrich Corporation (Stockholm, Sweden), and all the aqueous solutions were prepared using deionized [DI] water with a resistivity of 18.0 MΩ cm. Prior to the growth of ZnO NRs, the silicon substrate was ultrasonically cleaned sequentially in acetone, ethanol, and DI water, respectively, each step for 10 min, and then dried in the air. A seed layer was coated onto the substrate with the help of a spin coater spun at a rate of 2,200 rpm for 1 min. This procedure was repeated four times, as the seed layer acts as a preferential growth site during the growth process. In order to solidify the seed layer, the substrate was heated in air at a constant temperature of 250°C for 30 min. In the growth method, zinc nitrate hexahydrate [Zn(NO_3_)_2_⋅6H_2_O] (99.998%) and hexamethylenetetramine (C_6_H_12_N_4_) (99.998%) were mixed with equal molar concentration in DI water and kept under continuous magnetic stirring for 30 min to get a uniform growth solution. The seeded silicon substrate was then placed in the solution and was heated at 90°C for 6 h. After that, it was washed with DI water and dried in air.

One of the key and challenging steps in nanoscale mechanical testing is the manipulation and positing, with nanometer resolution and high throughput, of specimen at desired locations. After synthesis, the morphological analysis of the ZnO NRs and the required sample preparation for experiments were undertaken in a combined focused ion beam [FIB] and scanning electron microscope [SEM] instrument (FEI Co. Nova 600 Dual Beam, Ekerö, Sweden). In this study, we made a configuration of three-point bending with targeting holes by employing the FIB to etch the silicon wafer substrate, separate and pick up single NRs from the substrate, and place them lying over the grid. In order to eliminate the rod-substrate friction at the end points and prevent the NR from slipping during the nanoindentation experiment, a platinum film of approximately 50-nm thick was deposited at the end of the NRs using the precursor trimethyl (cyclopentadienyl) platinum [(C_5_H_5_)Pt(CH_3_)_3_]. Ga^+ ^ions were accelerated to a 5-keV and 10-pA operating current to perform the depositions.

A TriboIndenter (TI 950) system by Hysitron, Inc. (Minneapolis, MN, USA) was used to perform the tests on the single ZnO NR by directly indenting the center of the lying NRs. A three-sided, pyramidal Berkovich diamond indenter of 2 μm in diameter was used, with loads in the range of 0.2 to 20 μN. Despite the small size of the NRs, the imaging by scanning probe microscopy allowed us to estimate the position and place of indentation.

## Results and discussion

**Figure 1:**
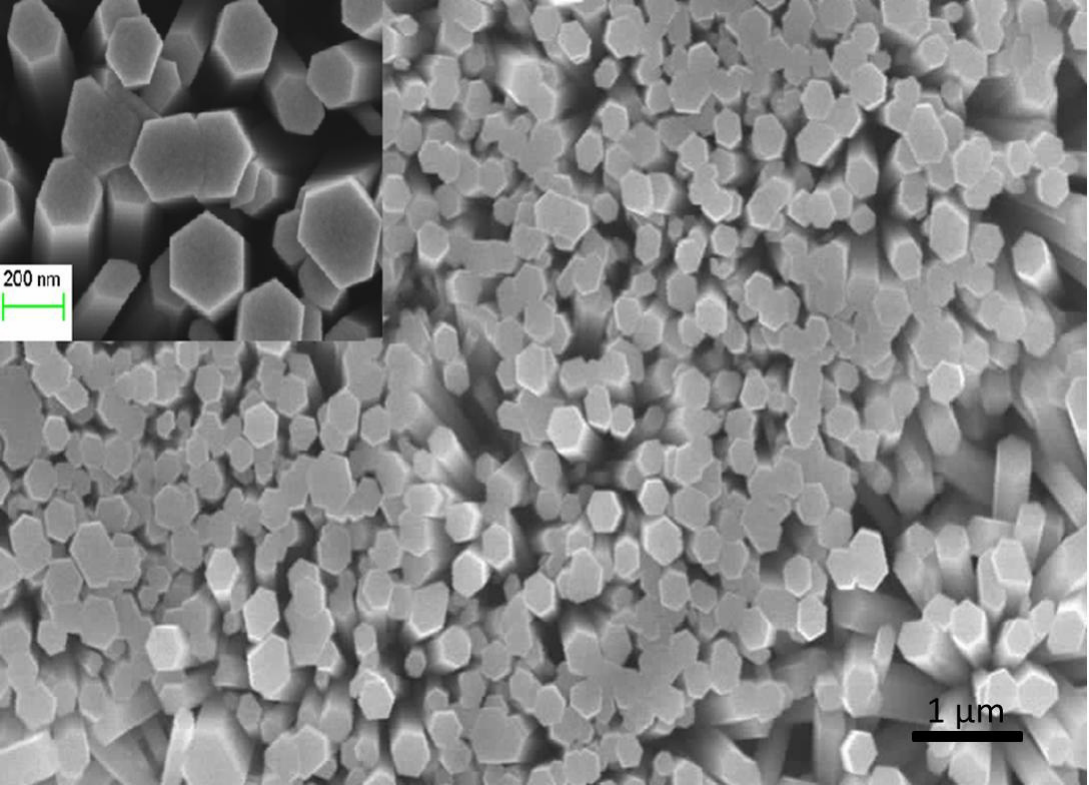
**SEM image showing the morphology of the as-grown ZnO NR arrays grown on silicon substrate**. Inset shows a magnified image.

The morphology of the as-grown ZnO NRs is shown in Figures 1 (top view) and 2 (side view). The ZnO NRs were found to be aligned vertically and uniformly distributed, with a well-defined hexagonal cross section. The average outer diameter and length of the ZnO NRs were about 200 nm and approximately 4 μm, respectively.

**Figure 2:**
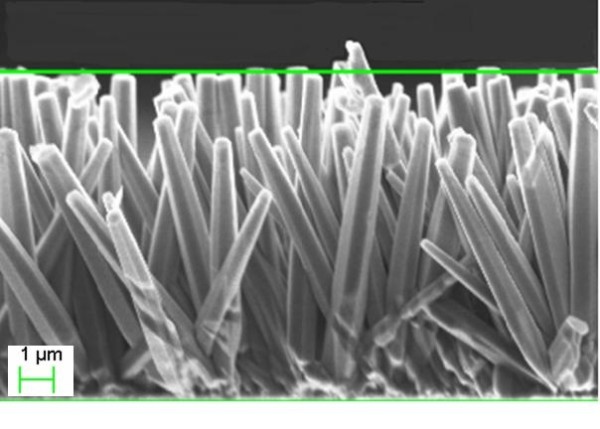
**SEM image showing morphology of as-grown ZnO NR arrays grown on silicon substrate (side view)**.

**Figure 3:**
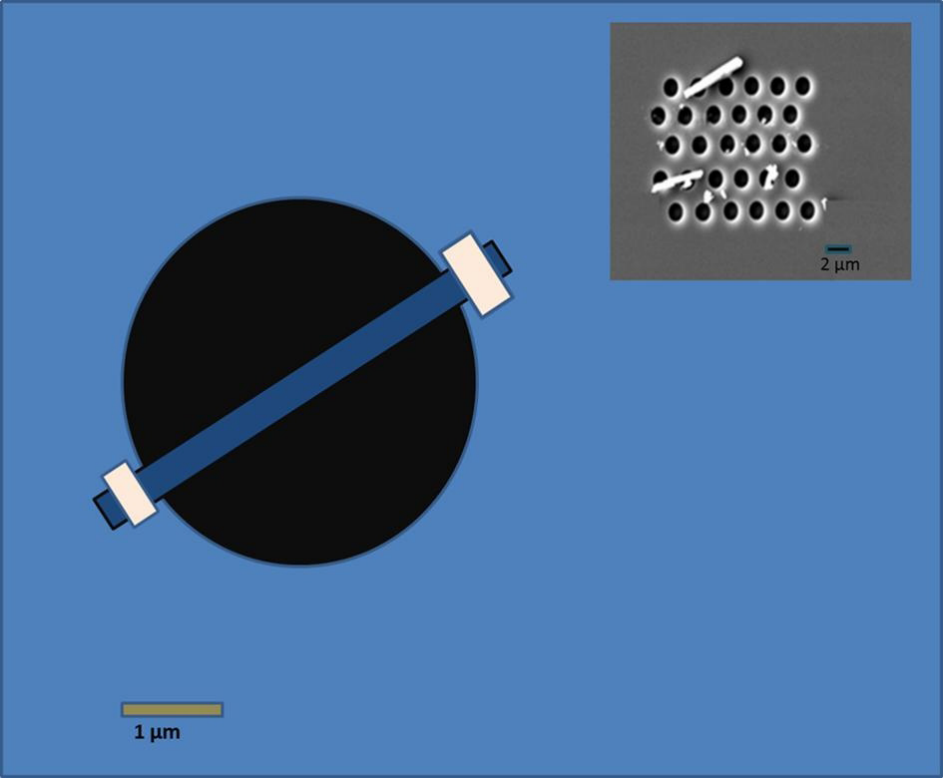
**Schematic diagram of a single ZnO NR**. Schematic diagram of a single ZnO NR with fixed ends placed at the trenched zone on the silicon wafer substrate. Inset shows a SEM image of typical suspended NRs on the silicon wafer substrate. The white particles are the residue of broken ZnO NRs.

In order to measure Young's modulus, we used a three-point bending configuration [21,22]. A schematic diagram showing a three-point bending configuration with a single horizontal ZnO NR is shown in Figure 3. The inset shows a SEM image of suspended NRs on the silicon wafer substrate; the material with the clear contrast is the residue of broken ZnO NRs.

**Figure 4:**
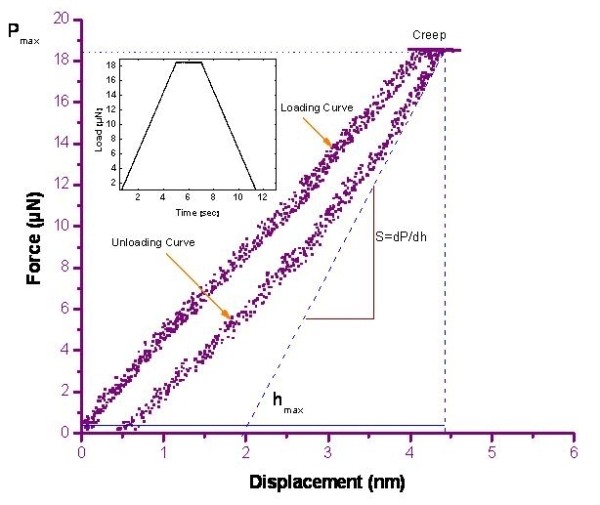
**Nanoindentation load-displacement curve**. Nanoindentation load-displacement curve with a peak indentation load-holding segment, showing creep at the peak load. The inset shows load-time profile.

After positioning the nanoindenter over a NR, the NR was loaded to a prescribed force and then unloaded in a force-controlled mode, and force-displacement curves were recorded. Several tests were repeatedly conducted in order to see the stability, and no permanent deformation of the NR was detected. Figure 4 displays the typical load-displacement curve of a single ZnO NR. This curve provides a mechanical fingerprint of the material's response to deformation as a function of the normal load. The three key parameters: the peak load [*P*_max_], the depth at peak load [*h*_max_], and the initial loading stiffness [*S*], are also indicated in the figure. In this study, Young's modulus of a single ZnO NR was evaluated using the Oliver-Pharr method [23] from the unloading curve because that portion of the curve is considered to be dominated by elastic recovery process in the materials. According to the Oliver-Pharr method during the load-unload cycle of the nanoindenter, the reduced elastic modulus (*E*_r_) of the sample is defined as:

(1) $$E_{\text{r}}=\frac{\sqrt{\pi }}{2\beta \sqrt{A}}*S.$$

This equation can be written in terms of the combined reduced elastic modulus specimen/indenter (*E*_r_):

(2) $$\frac{1}{E_{\text{r}}}=\frac{\left(1-\upsilon_{\text{i}}^{2}\right)}{E_{\text{i}}}+\frac{\left(1-\upsilon_{\text{s}}^{2}\right)}{E_{\text{s}}},$$

where *A *is the area of the indentation at the contact depth *h*_c_, *S *= dp/dh is the initial unloading curve at maximum load or stiffness which is proportional to the modulus, and *E*_s_, *ν*_s_, *E*_i_, and *ν*_i _are the elastic modulus and Poisson's ratio of the specimen and the indenter, respectively. The parameter *β *in Equation 1 is a constant that depends on the geometry of the indenter; for example, for a conical indenter, it has a value of 1. For an ideally sharp Berkovich indenter, the projected area (*A*) and the contact depth (*h*_c_) are related ideally as

(3) $$A=24.5h_{\text{c}}^{2}\;\text{and}\;\;h_{\text{c}}=h_{\text{max}}-\frac{\varepsilon P_{\text{max}}}{S},$$

here *h*_max _is the maximum displacement at the peak load, *P*_max _is defined as the maximum load, and *ε *is the geometric constant which depends on the shape of indenters. For conical indenters, their value is 0.72. Substituting the appropriate values for different variables *β *= 1, Poisson's ratio (*ν*_i_) for the diamond indenter = 0.07, Poisson's ratio (*ν*_s_) for ZnO = 0.358, Young's modulus for the Berkovich diamond indenter (*E*_i_) is approximately 1,140 GPa, and calculated Young's modulus (*E*_s_) of the single ZnO NR results in approximately 800 GPa. During the experiment, we assumed that Poisson's ratio of the NR is unchanged due to the fact that both ends of the NR remain in the same place even after the test is conducted. To compare our calculated results to those of previous studies, Young's modulus of pure ZnO films was reported in the range of 64 to 128 GPa [24,25] and a typical bulk ZnO of about 144 GPa [26,27]. There are many hypotheses to explain the origin of discrepancy in the elastic modulus in NRs such as the Hall-Petch effect [28,29], according to which the smaller grain size of the material has a higher material strength. According to another theory, the surface-to-volume ratio increases the surface unsaturated atomic states, which are believed to affect the dislocation generation and motion in a different way to the locked atoms inside that lattice, thus affecting the mechanical behavior of 1D nanomaterials [30]. Other reasons attributed to larger calculated values of the elastic modulus can be the indentation pile-up, the indent size effect, and the elastic recovery [31].

In our indentation experiments, creep of approximately 0.5 nm can also be observed. The creep occurs during the 2-s holding segment of the nanoindentation, as shown in the inset of Figure 4. Creep is a form of mechanical degradation, and it is best assessed under load-control nanoindentations. The creep mechanism is still unknown, but the elastic deformation as a result of the diffusion and motion of the atoms or motion of the dislocations seems to play an important role in developing creep during the peak indentation load-holding segment [20]. Generally, for metals, ceramics, and semiconductors, time-dependent deformation occurs at high temperatures, but in our case, by performing indentation testing on a single horizontal ZnO NR, creep occurs even at room temperature. The possible reason can be related to quantum confinement: the surface atoms can move more easily than the bulk where the atoms are fully locked in the lattice [32]. This condition is favorable for the making and motion of dislocations which makes it much easier underneath the indenter, leading to the indentation displacement.

## Conclusion

In summary, we report the experimental finding of the elastic modulus of a single ZnO NR using the nanoindentation technique to perform a three-point bending. The elastic modulus obtained from direct nanoindentation measurement is in the range of 800 GPa. We also observed creep during our experiments, probably due to the diffusion and motion of the atoms and/or dislocations.

## Competing interests

The authors declare that they have no competing interests.

## Authors' contributions

MYS, IH, NB, ON, and MW initiated the presented study, measured the elastic modulus of a single horizontal ZnO nanorod grown by low-temperature hydrothermal chemical process on silicon substrates, and wrote the manuscript. EB provided all the measured results from nanoindentation measurements. All the authors participated in the revision and approval of the manuscript.
